# A Validated TLC-Densitometric Method for the Determination of Mesterolone in Bulk Material and in Tablets

**DOI:** 10.1155/2015/230104

**Published:** 2015-12-31

**Authors:** Małgorzata Dołowy, Alina Pyka-Pająk, Katarzyna Filip, Joanna Zagrodzka

**Affiliations:** ^1^Department of Analytical Chemistry, School of Pharmacy and the Division of Laboratory Medicine, Medical University of Silesia in Katowice, 4 Jagiellońska, 41-200 Sosnowiec, Poland; ^2^R&D Analytical Chemistry Department, Pharmaceutical Research Institute, 8 Rydygiera, 01-793 Warsaw, Poland

## Abstract

Mesterolone is a synthetic androgenic steroid indicating a weak anabolic activity. A new, simple in use, and economical TLC-densitometric method in normal phase system (NP-TLC) has been developed and validated for the identification and quantitative determination of mesterolone in bulk drug and in tablet formulation. NP-TLC analysis was performed on aluminium plates precoated with silica gel 60F_254_ as the stationary phase using chloroform-acetone (40 : 10, v/v) as mobile phase. Densitometric analysis was carried out at *λ* = 745 nm after staining with phosphomolybdic acid. These conditions were found to give visible (dark blue) spot and sharp peak, respectively, for mesterolone at *R*_*F*_  0.75 ± 0.02 and enabled satisfactory separation of mesterolone from its related substance (potential impurity). The proposed NP-TLC-densitometric method was validated for specificity, linearity, precision, accuracy, robustness, and sensitivity according to ICH guideline and other validation requirements. The limit of detection (LOD) and limit of quantification (LOQ) were 61.0 ng·spot^−1^ and 184.0 ng·spot^−1^, respectively. The percent content of mesterolone in marketed tablet formulation was found to be 99.40% of label claim. The developed TLC-densitometric method can be successfully used in quality control of mesterolone in bulk material and also tablet formulation.

## 1. Introduction

**Figure 1:**
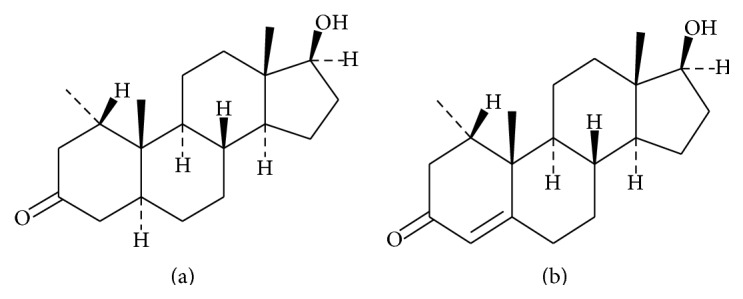
Chemical structure of mesterolone (a) and its impurity (b).

Mesterolone (Figure 1(a)) is a synthetic androgenic steroid indicating a weak anabolic activity. It is clinically used in the treatment of hypogonadism in the case of declining of physical and mental capacity caused by the deficiency of androgens and also in male infertility [1]. This compound is an active ingredient of different pharmaceutical preparations available in drug markets in form of tablets containing generally from 25 to 50 mg of mesterolone per tablet. In addition to its medical uses, mesterolone, similarly, like other anabolic-androgenic steroids (AAS), has been widely applied in sport (e.g., by athletes) in order to improve athletes' performance [2]. Mesterolone belongs to the list of the prohibited substances by WADA (World Anti-Doping Agency). Despite the restriction on the use of AAS in sport and their various side effects, mesterolone and its related substances can be illicitly marketed and distributed in form of different dietary supplements [3, 4]. Therefore, there is a crucial need to develop a method for the quality control of commercially available pharmaceutical formulations containing mesterolone and also preparations which are coming from illegal source (e.g., from black market). Numerous analytical methods, such as UV-Vis spectrophotometry, gas chromatography (GC), and high-performance liquid chromatography (HPLC) with ultraviolet-visible or mass spectrometry detection, have been successfully developed for the analysis of mesterolone in a variety of biological and also pharmaceutical samples [5–12]. Currently, among different advanced techniques, ^1^HNMR spectroscopy could be a good tool for the determination of pharmacologically active substances, such as examined mesterolone and its analogues in dietary supplements that have been illegally distributed [13]. Literature review on the methods for the analysis of steroids indicates that, of many instrumental methods, thin-layer chromatography (TLC) combined with densitometry can be used for the determination of different steroids because of its simplicity and low detection limit up to nanograms/spot [14–17]. Thus, it is comparable with other modern but more expensive chromatographic methods.

According to the best of the authors' knowledge, there is no report for the development of TLC-densitometric method for the quantification of mesterolone in tablet dosage form so far. Therefore, in the present study, a simple, sensitive, accurate, and precise NP-TLC method coupled with densitometry has been developed for the determination of mesterolone in the bulk and also tablet formulation. The suitability of TLC-densitometry for the quantitative determination of mesterolone was proved by validation in accordance with the requirements of ICH guidelines (International Conference on Harmonization) [18] and with respect to other recommendations which are reported by Ferenczi-Fodor and Konieczka and coworkers, respectively [19–21].

## 2. Experimental

### 2.1. Chemicals

Mesterolone European Pharmacopoeia reference standard, Figure 1(a) (1*α*-methylandrostan-17*β*-ol-3-one, CAS number 1424-00-6), and its impurity A, Figure 1(b) (17*β*-hydroxy-1*α*-methylandrost-4-en-3-one, CAS number 604-26-2), were procured from Sigma-Aldrich (St. Louis, MO, USA). The components of mobile phase, chloroform and acetone with chemical grade, were from POCh, Gliwice, Poland. Ethanolic solution of sulphuric acid at concentration 10 [%, w/w] (POCh, Gliwice, Poland) and also 10 [%, w/w] solution of phosphomolybdic acid in ethanol (POCh, Gliwice, Poland) was used as the visualizing reagents. Ethanol (96%), its chemical grade (POCh, Gliwice, Poland), was applied to prepare the two examined visualizing reagents.

Pharmaceutical formulation containing 25 mg of examined mesterolone in tablet was from local drug market.

### 2.2. Chromatographic State and Apparatus

Spectrodensitometric scanning was done using a Camag TLC Scanner 3 (Muttenz, Switzerland) and WinCATS 1.4.2 software. All measurements were performed in the reflectance/absorbance mode. The source of the light was deuterium and wolfram lamp.

Plate development was carried out in twin trough chromatographic chambers: 10 cm × 20 cm (#0.222.5221, Camag, Muttenz, Switzerland). Chromatographic plates, 10 cm × 10 cm, were cut from 20 cm × 20 cm (E. Merck, Germany) for NP-TLC analysis: aluminium plates precoated with silica gel 60F_254_ (Art. 1.05554), aluminium plates precoated with silica gel 60 (Art. 1.05553), and aluminium plates precoated with a mixture of silica gel 60 and Kieselguhr F_254_ (Art. 1.05567) were examined. Micropipettes (5 *µ*L, Camag, Muttenz, Switzerland) have been used for the spotting of samples and standard on TLC plates in quantity of 5 *µ*L in each case. The plates were activated at 100°C before chromatography. Chromatographic process was carried out at room temperature (23 ± 2°C). The TLC plates were developed up to a distance of 80 mm using 50 mL of eluent consisting of chloroform and acetone (40 : 10, v/v). This mobile phase was selected from others on the basis of literature review and our previous studies on the determination of different classes of steroids including anabolic-androgenic steroids [14–17]. Among various TLC systems which are widely recommended for analysis of AAS, such as mesterolone, the best are those which are consisted of chloroform as the main component of mobile phase and silica gel 60F_254_ TLC plates. In our preliminary study we have checked the usefulness of various mobile phases which consisted of chloroform, like, for example, chloroform and methanol (48.5 : 1.5, v/v), chloroform-ethanol-water (47 : 3 : 0.25, v/v/v),* n*-hexane-chloroform-methanol (10 : 30 : 2.5, v/v/v), chloroform-acetone, and NP-TLC plates and also methanol-water mixture in different volume composition and RP-TLC plates. The above mentioned mobile phases could be optimal for TLC study of pure mesterolone only (its *R*_*F*_ is placed in the range of 0.3–0.8). In order to investigate this compound in the presence of its potential degradation product (impurity A) there is a need to modify some of these mobile phases to achieve the optimal *R*_*F*_ value of both compounds and to obtain satisfactory resolution of them. We observed that addition of acetone to mobile phase which consisted of chloroform only can improve the resolution of studied steroids. Finally, this mobile was used for further optimization of applied TLC system and chloroform-acetone in volume composition of 40 : 10 was found to be the best.

The optimized chamber saturation time was 20 min. After development plates were dried in current of air during 24 h. Phosphomolybdic acid (PMA) was applied to visualize the examined compound. Developed plates were dipped into 10% phosphomolybdic acid or 10% sulphuric acid, respectively, for 1-2 minutes and next heated at 120°C for 15 min in a preheated oven. Camag TLC Scanner 3 was used for scanning at *λ* = 745 nm. The slit dimension was kept at 8.00 mm × 0.40 mm, Macro, the scanning speed was 20 mm·s^−1^, and the data resolution was 100 *µ*m·step^−1^.

### 2.3. Preparation of Standard Solutions of Mesterolone and Its Related Substance

Standard stock solutions of mesterolone and its impurity A have been prepared separately in methanol (POCh, Gliwice, Poland) at concentration of 1 mg·mL^−1^. Accurately weighed 10 mg of each mesterolone standard and its impurity was dissolved in 10 mL of methanol. Stock solution of mesterolone was further diluted in methanol to obtain working standard solutions of various concentrations and stored at 2–8°C until use.

## 3. Validation of the Method

Validation of the proposed TLC method was carried out with respect to the following parameters [18–21].

### 3.1. Specificity

The specificity of the method was ascertained by analyzing standard solution of mesterolone, its related substance (impurity A), and also sample solution of mesterolone extracted from tablets.

### 3.2. Linearity Study

The linearity of proposed TLC method was evaluated by analysis of seven standard solutions of mesterolone at concentration of 0.02, 0.04, 0.08, 0.12, 0.16, 0.20, and 0.24 mg·mL^−1^. The solutions (5 *µ*L) were applied to the same plate (Art. 1.05554). The plates were developed using mobile phase, chloroform-acetone in volume composition 40 : 10, and scanned at *λ* = 745 nm after visualization of obtained bands with use of PMA (phosphomolybdic acid). The experiments were performed in six different analyses.

### 3.3. Limit of Detection (LOD) and Limit of Quantification (LOQ)

To estimate the sensitivity of proposed method (LODs and LOQs), the procedure based on the standard deviation of the response and the slope of specific calibration plot was applied. A specific calibration curve was studied using samples containing mesterolone in the range of the detection limit, namely, 400, 500, and 600 ng·spot^−1^. The experiments were performed in six different analyses.

Limit of detection (LOD) was calculated according to the following formula:

(1) $$\begin{array}{c} LOD=\frac{3.3\times \sigma }{S}. \end{array}$$

Limit of quantification (LOQ) was estimated by use of the following equation:

(2) $$\begin{array}{c} LOQ=\frac{10\times \sigma }{S}, \end{array}$$

where *S* is the slope of the specific calibration curve and *σ* is the standard deviation of the response.

To estimate the standard deviation (*σ*), the following parameters have been determined:

(i) Standard deviation of intercept of specific regression plot (*S*_*a*_).
(ii) Residual standard deviation of specific regression plot (*S*_*xy*_).

Moreover, to obtain reliable LOD and LOQ values, the correctness of the designated detection limit (LOD) was estimated by comparison of the obtained results with the following conditions [21]:

(3) 10×LOD>C,LOD<C,LOQ=3×LOD,

where LOD is the limit of detection, LOQ is the limit of quantification, and *C* is the concentration of analyzed mesterolone in the standard sample.

### 3.4. Accuracy

Recovery experiments of the mesterolone at different levels in examined pharmaceutical formulation were conducted to check the accuracy of the method. Known amounts of standard substance in the low (80%), medium (100%), and high (120%) level of tested content were added to the sample (powdered tablets), and the tablets were extracted and analyzed under the optimized conditions. Each experiment was performed three times at each level.

### 3.5. Precision

The precision of the method (RSD %) was assessed by repeatability (intraday precision) and intermediate precision studies (interday precision). The intraday and interday precision were performed by analysis of three different concentration levels of mesterolone: 200 ng·spot^−1^, 300 ng·spot^−1^, and 400 ng·spot^−1^ under the same operating conditions over a short interval of time (the same day). The interday variation was assessed by studying three sample concentrations by an analyst who performed the analysis over a period of two weeks. To determine the precision of the procedure, the concentrations were prepared independently and experiments were performed in three different analyses.

### 3.6. Robustness Study

Robustness was estimated by changing different chromatographic conditions in proposed procedure. The effect of small, deliberate chromatographic conditions on the results (peak area of examined mesterolone) was described:

- Mobile phase volume (varied ±5%).
- Time of activation of the plates at 100 ± 2°C for 20, 30, and 40 minutes before analysis.
- Development distance (±50 mm).
- Time from spotting to chromatography (±10 min).
- Duration of saturation (±5 min).

### 3.7. Analysis of Marketed Formulation of Mesterolone

To determine the mesterolone content in pharmaceutical formulation, ten tablets containing label claim 25 mg/tablet were crushed for 20 min with a speed equal to 5000 rpm using IKA Ultra-Turrax Tube Drive Workstation with BMT-20-S Tube for grinding with balls of stainless steel. Amount of obtained powder equivalent to one tablet was extracted with 10 mL of methanol for 15 min with a speed equal to 5000 rpm. The resulting solution was filtered through a medium-density filter and the filtrate was diluted with methanol to concentration of 1 mg·mL^−1^. For the further drug content analysis the solution at concentrations of examined mesterolone equal to 0.10 mg·mL^−1^ was used. 5 *µ*L of filtrate was spotted onto the chromatographic plates (Art. 1.05554) and then chromatographically analyzed in combination with densitometry (using chloroform-acetone 40 : 10 and PMA as visualizing reagent) for quantitative determination of mesterolone in pharmaceutical preparation. This analysis was repeated six times.

### 3.8. Statistical Evaluation of Results

Statistical evaluation of the obtained results and also the similarity analysis were prepared by Statistica v 10.0 PL (StatSoft, Kraków, Poland).

## 4. Results and Discussion

### 4.1. Optimization of Chromatographic Conditions

To obtain the desired *R*_*F*_ value range (0.3–0.8) of examined mesterolone various mobile phases containing chloroform and acetone in the volume compositions, 50 : 0; 45 : 5; 40 : 10; 35 : 15; 30 : 20; 25 : 25, and 0 : 50, and three types of aluminium TLC plates from E. Merck, precoated with silica gel 60 (Art. 1.05553), silica gel 60F_254_ (Art. 1.05554), and silica gel 60 and Kieselguhr F_254_ (Art. 1.05567) were applied. Although based on the literature data, various visualization modes, without visualizing agent, with the use of sulphuric acid, and using phosphomolybdic acid, were estimated. The results of obtained *R*_*F*_ values confirm that, of all mobile phases, the phase which consisted of chloroform-acetone 40 : 10 is suitable for identification of mesterolone on three applied chromatographic plates: Art. 1.05553, Art. 1.05554, and Art. 1.05567. The peaks obtained using this solvent system onto all TLC plates applied in this experiment are sharp and compact. Finally, this mobile phase and chromatographic plates (Art. 1.05554) were used in further study. Two different visualizing reagents like sulphuric acid and phosphomolybdic acid (PMA) were tried, while the best results were achieved with PMA. Derivatization with PMA gave more colourful and compact spots (dark blue on the green background) and sharp peaks, respectively, in comparison with that obtained using sulphuric acid or without derivatizing reagent. The suitability of proposed chromatographic system for the determination of mesterolone in both bulk drug and tablet formulation is shown in Table 1.

**Figure 2:**
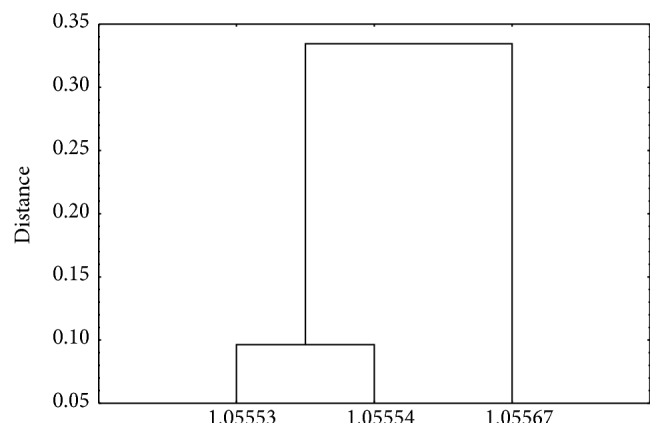
Dendrogram of the similarity analysis: *R*_*F*_ values of mesterolone separated on chromatographic plates Art. 1.05567, Art. 1.05554, and Art. 1.05553 using chloroform-acetone 40 : 10 (v/v) and visualized by phosphomolybdic acid (PMA).

Similarity analysis of *R*_*F*_ values in Figure 2 indicates that, of all TLC plates used in detection of mesterolone by chloroform-acetone (40 : 10, v/v), the biggest similarity in *R*_*F*_ value indicates Art. 1.05553 and also Art. 1.05554 (the smallest distance between both is observed on dendrogram in Figure 2). Thus, the chromatographic plates precoated with silica gel 60 (Art. 1.05553) can be alternatively used to those applied in this study which are coated with silica gel 60F_254_ (Art. 1.05554).

### 4.2. Specificity and Selectivity

**Figure 3:**
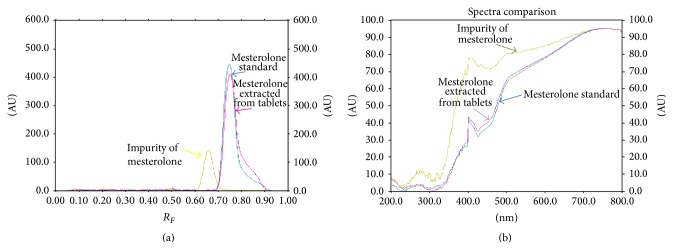
Comparison of TLC densitograms (a) and spectrodensitograms (b) of examined mesterolone standard, mesterolone extracted from tablets, and its impurity.

To verify the specificity of the developed method, drug sample (extract from tablets) and mesterolone standard were simultaneously analyzed under chromatographic conditions which have been found to be optimal, such as chromatographic plates Art. 1.05554, chloroform-acetone in volume composition of 40 : 10, and PMA as derivatizing reagent. Densitometric analysis was performed at *λ* = 745 nm. The spots of mesterolone in examined pharmaceutical formulation (tablets) were confirmed by comparing *R*_*F*_ values and also spectra (Figure 3) with those of standard. Both peaks coming from standard and also from sample (tablets) appeared at *R*_*F*_ = 0.75 ± 0.02 (Figure 3(a)). Comparison of spectra obtained for standard and sample solution indicate also the identity of sample with its standard (Figure 3(b)).

In order to check the selectivity and thus ability of proposed TLC-densitometric method using prederivatization of spots by PMA to identify mesterolone in the presence of its related substance (potential interference, like, e.g., impurity A), the comparison of densitogram and spectrodensitogram of standard, impurity, and extract from tablets was done. Satisfactory resolution of mesterolone (active ingredient) from its impurity confirmed the separation factors, Δ*R*_*F*_, *R*_*S*_, and *α* (listed in Table 2), which have been calculated on the basis of densitogram placed in Figure 3. Separation factors for examined pair of mesterolone and its impurity are equal to *α* = 1.54, *R*_*S*_ = 1.05 (higher than 1), and Δ*R*_*F*_ ≥ 0.05 (Δ*R*_*F*_ = 0.09). The three resolution parameters show good separation of examined active substance (mesterolone) from its related substance (impurity A).

**Figure 4:**
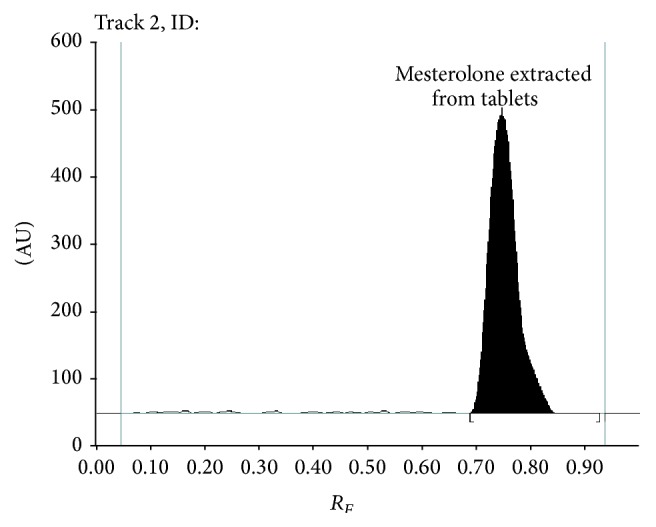
Densitogram of mesterolone extracted from tablets scanned at *λ* = 745 nm.

In addition to this, TLC densitogram of mesterolone extracted from tablets produced under described chromatographic conditions (Figure 4) indicates that there is no interference from any of the excipients which are present in sample.

### 4.3. Linearity and Range

**Figure 5:**
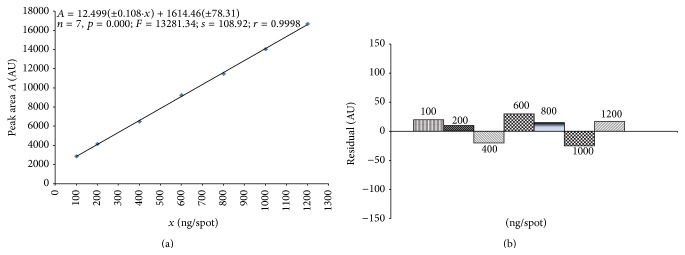
Calibration plot (a) and plot of residuals (b) for mesterolone in the linear working range.

Linearity was estimated by applying stock solutions of mesterolone onto TLC plates (Art. 1.05554) in the range of 100–1200 ng·spot^−1^. Peak area (*A*) versus corresponding amount of mesterolone (*x*) was used to construct calibration plot (Figure 5(a)). Each amount was applied six times and the plates were developed under optimized mobile phase, derivatized by PMA, and scanned at *λ* = 745 nm. Linear regression equation was found to be *A* = 12.499(±0.108) · *x* + 1614.46(±78.31). The regression coefficient (*r* = 0.9998), significance level (*p* = 0.000), and standard error of estimate *s* = 108.92 indicate good correlation between peak area and mesterolone concentration. Moreover, residual linearity test was used to check the linearity of obtained calibration plot (Figure 5(b)). Residual linearity test confirms also linearity of proposed method because the residual values are placed above and below zero line (Figure 5(b)).

### 4.4. Limit of Detection and Limit of Quantification (LOD and LOQ)

Detection limit and quantification limit were calculated by the method described in Experimental. The average values of LOD and LOQ were found to be 61.0 ng·spot^−1^ and 184.0 ng·spot^−1^. This indicates that sensitivity of proposed TLC-densitometry method is adequate to determine mesterolone in its pharmaceutical formulation containing generally from 25 to 50 mg per tablet and it is comparable with other chromatographic methods.

### 4.5. Accuracy

The accuracy of the method was carried out by measurement of recovery mesterolone standard corresponding to 80%, 100%, and 120% of declared content which was added to the powdered tablets containing known amount of mesterolone. The samples were analyzed for content determination in optimized chromatographic conditions. Summary of recovery data is given in Table 3. Recoveries of 99.43 ÷ 100.92% and the low value of % relative standard deviation (RSD < 2) indicate the accuracy of this method.

### 4.6. Precision

The intraday and interday precision of developed method were expressed in terms of % RSD. The results presented in Table 4 show mean % RSD in the range of 0.69 ÷ 1.67 in the case of intraday precision and from 1.12 to 1.79 for interday precision. According to ICH guideline the obtained % RSD values less than 2 confirm good precision of method.

### 4.7. Robustness of the Method

The standard deviation of peak areas measured for each of five parameters which have been changed in conducted experiment in order to check the robustness of applied method is placed in the range of 0.45 to 1.12 (Table 5). The low value of % RSD (<2) indicates the reliability of proposed TLC-densitometric method during its normal use.

### 4.8. Content Determination in Commercial Dosage Form (Tablets)

Sample chromatogram of mesterolone extracted from tablets shows single spot at *R*_*F*_ = 0.75 ± 0.02 (Figure 4). No interference from the excipients and related substances occurs in tablet formulation analyzed in this pharmaceutical preparation, like, for example, examined impurity A (17*β*-hydroxy-1*α*-methylandrost-4-en-3-one) which was observed. Identity of mesterolone standard and that coming from tablets was confirmed by comparison of *R*_*F*_ values and also their spectra. The very good correspondence between retardation factor (*R*_*F*_) and also spectrodensitograms for mesterolone standard and sample was stated. In both cases the absorption maximum (*λ*_max_) is equal to 745 nm and *R*_*F*_ is 0.75 for standard and sample. Summary of TLC-densitometric analysis of tablet dosage form is highlighted in Table 6. Statistical data concerning the results of quantitative determination of mesterolone in the six repeated different analyses of examined pharmaceutical preparation presented in Table 6 indicate that the average amount of mesterolone in pharmaceutical preparation determined by TLC-densitometric method is equal to 99.40% in relation to the amount of mesterolone declared by the manufacturer and it is in agreement with the percent content required by European Pharmacopoeia for mesterolone as active ingredient [22]. The value of % RSD less than 2 indicates the suitability of proposed method for routine analysis of mesterolone in tablet dosage coming from legal source and also that which can be illegally distributed as dietary supplements.

## 5. Conclusion

A new, simple, and economic NP-TLC method combined with densitometry has been developed and validated for the identification and also quantification of mesterolone in bulk material and in its tablet dosage form. Statistical analysis of obtained results indicates that the method is specific, precise, accurate, robust, and sensitive. The method is suitable for analyzing of mesterolone in bulk material and tablet formulation without any interference from additives present in pharmaceutical formulation. It can be concluded that the elaborated TLC-densitometric method may be used in the quality control of pharmaceutical formulation containing mesterolone as well as dietary supplements applied by athletes (in doping analysis).

## Figures and Tables

**Table 1:** Chromatographic characteristic of mesterolone analyzed using different NP-TLC plates and chloroform-acetone (40 : 10, v/v) as mobile phase.

Mobile phase	Chromatographic plates	*R*_*F*_	Visualization mode		
			Densitometric analysis without the use of visualizing agent	Densitometric analysis after derivatizing with 10% sulphuric acid	Densitometric analysis after derivatizing with 10% phosphomolybdic acid (PMA)
			*λ*_max⁡_ [nm]	*λ*_max⁡_ [nm]	*λ*_max⁡_ [nm]
Chloroform-acetone 40 : 10 (v/v)	Silica gel 60 Art. 1.05553 (E. Merck)	0.66 ± 0.01	200	467	745
	Silica gel 60F_254_ Art. 1.05554 (E. Merck)	0.75 ± 0.02	200	467	745
	Silica gel 60/Kieselguhr F_254_ Art. 1.05567 (E. Merck)	0.87 ± 0.01	248	468	745

**Table 2:** Results of the resolution of mesterolone and its impurity on TLC plates (Art. 1.05554) developed using mobile phase: chloroform-acetone (40 : 10, v/v).

Parameter	Mesterolone	Impurity A
*R*_*F*_	0.75	0.66
*α*	1.54	
*R*_*S*_	1.05	
Δ*R*_*F*_	0.09	

**Table 3:** Recovery studies.

Drug	Initial amount of mesterolone [ng·spot^−1^]	Amount of standard mesterolone added [%]	Amount of standard mesterolone added [ng·spot^−1^]	Amount recovered	% recovery (*n* = 3)	Mean % RSD (*n* = 3)
Mesterolone (tablet)	300.0	80	240.0	239.51	99.80	
	300.0	80	240.0	239.25	99.69	0.11
	300.0	80	240.0	238.97	99.57	
	300.0	100	300.0	302.76	100.92	
	300.0	100	300.0	298.32	99.44	0.82
	300.0	100	300.0	298.65	99.55	
	300.0	120	360.0	357.98	99.43	
	300.0	120	360.0	360.96	100.27	0.48
	300.0	120	360.0	358.10	99.47	

RSD, relative standard deviation.

**Table 4:** Intraday and interday precision of developed method.

Drug	Initial amount of mesterolone [ng·spot^−1^]	Intraday		Interday	
		Amount of mesterolone found	Mean % RSD (*n* = 3)	Amount of mesterolone found (*n* = 3)	Mean % RSD (*n* = 3)
Mesterolone (tablet)	200.0	197.34	0.69	196.34	1.25
	200.0	200.01		192.01	
	200.0	198.15		196.15	
	300.0	288.35	1.67	294.25	1.12
	300.0	291.45		289.85	
	300.0	297.89		287.89	
	400.0	387.86	1.12	397.16	1.79
	400.0	396.54		391.44	
	400.0	391.32		383.22	

RSD, relative standard deviation.

**Table 5:** Robustness of the proposed method (*n* = 3).

Parameter	% RSD of peak area
Amount of mobile phase volume (varied ±5%)	0.56
Time of activation of the plates at 100 ± 2°C for 20, 30, and 40 minutes before analysis	0.62
Development distance (±50 mm)	0.45
Time from spotting to chromatography (±10 min)	0.58
Duration of saturation (±5 min)	1.12

RSD, relative standard deviation.

**Table 6:** Results from assay of mesterolone in tablets [mg·tablet^−1^] (*n* = 6).

Number	Amount detected
1	25.12
2	24.76
3	24.32
4	25.01
5	24.94
6	24.93
Average amount found	24.85
Label claim	25.00
Standard deviation (SD)	0.283
% relative standard deviation (RSD)	1.14
Percentage of label claim	99.40
